# Potential Biomarkers and Underlying Pathogenesis of *Mycoplasma synoviae* Infection: Insights from Metabolomics Analysis

**DOI:** 10.3390/microorganisms13112427

**Published:** 2025-10-23

**Authors:** Xiaona Wei, Mengyao Sun, Kuan Zhang, Jing Wen, Zhuanqiang Yan, Yangxue Liu, Lianxiang Wang

**Affiliations:** 1 School of Life Sciences, Zhengzhou University, Zhengzhou 450001, China; 2 Wen’s Group Academy, Wen’s Foodstuffs Group Co., Ltd., Yunfu 527400, China

**Keywords:** *Mycoplasma synoviae*, untargeted metabolomics, pathogenesis, virulence, significantly differentially abundant metabolites

## Abstract

*Mycoplasma synoviae* (MS) is a prominent poultry pathogen that has caused considerable economic pressure on the poultry industry. Although we have a good understanding of MS infection, research is still lacking on the pathogenicity of MS and host-MS interactions, especially the metabolic basis of MS infection. In this study, a lethal MS strain ZX313 was identified. Then, untargeted metabolomic analysis was performed on the plasma of 18 SPF chickens infected with the ZX313 strain and the low-virulence strain SD2. A total of 699 and 720 significantly differentially abundant metabolites (SDMs) were detected after ZX313 and SD2 infection, respectively, among which 95 and 116 SDMs were group-specific. Metabolic pathway enrichment analysis revealed that MS infection significantly disturbed host amino acid, nucleotide and lipid metabolism. Moreover, the differential expression of amino acid metabolism in different virulence groups may be related to the severity of the disease and the pathogenicity of MS. A total of 20 plasma metabolites were identified to exhibit a significant correlation with disease severity, with an area under the curve of 0.986. These findings demonstrate that the host’s systemic metabolism undergoes significant changes following MS infection, providing valuable references for elucidating infection-related metabolic alterations and their association with disease severity.

## 1. Introduction

*Mycoplasma synoviae* (MS) is an important pathogenic mycoplasma worldwide that causes joint and foot pad swelling, as well as upper respiratory disease, in infected chickens. MS has the potential to spread by means of direct contact and vertically via fertilized eggs [1,2]. Although studies have reported variable pathogenicity among different MS isolates, MS infection alone causing avian mortality is extremely rare [3]. Moreover, fewer in-depth studies or explorations have been conducted regarding the mechanisms underlying the pathogenic differences among various virulent MS strains as well as the factors associated with the degree of disease severity.

The pathogenesis of MS is complex and multifactorial. MS infection of chicken synovial sheath cells triggers an inflammatory cascade, manifested by the vigorous upregulation of relevant cytokines and induction of macrophage chemotaxis [4,5]. Ma et al. demonstrated that the expression of heat shock proteins and genes related to inflammatory factors was associated with MS pathogenicity [6]. According to integrated transcriptomic and proteomic analyses, Liu et al. reported that a multitude of factors related to proliferation, apoptosis, inflammation, proangiogenic, antiangiogenic, and arthritis are potentially implicated in the pathogenesis of MS [7]. Furthermore, MS infection induces splenocyte apoptosis and oxidative stress, compromises tissue integrity, and activates the NF-κB/MAPK-mediated inflammatory cascade, thereby potentiating the pathological progression [8]. Based on the above, one of the most prominent characteristics of mycoplasma infection is the induction of a strong inflammatory response.

Metabolites are the intermediary or terminal substances of diverse metabolic processes. The aberrant manifestations of metabolites offer immediate and direct insights into the host’s physiological and biochemical responses elicited by distinct external stimuli, thereby constituting a crucial aspect of understanding the host–pathogen interaction dynamics and overall biological adaptation mechanisms [9]. Metabolomics is the systematic analysis of these functional small molecules and has been used to study different infectious or noninfectious diseases [10,11,12,13]. Infection with MS causes a severe inflammatory response [5,8] and may alter metabolites associated with inflammation, with changes in the abundance of these metabolites potentially correlated with the severity of disease occurrence. In studies on mutations of live MS vaccine strains, mutations in the live vaccine strains can reverse their own metabolism, and this metabolic reversal exhibits a certain correlation with the pathogenicity of the strains [14]. A recent study has shown that after infecting laying hens, MS strains with a reproductive system tropism colonize the oviduct and cause pathological damage; their infection induces multiple metabolic disorders in SPF laying hens, disrupts signaling pathways, and subsequently results in host cell damage [15].

In this study, two isolated strains of MS, ZX313 and SD2, were identified and verified for their different pathogenicity to SPF chickens. Plasma samples from chickens infected or not infected with different MS virulence strains were collected and subjected to untargeted metabolome analysis to assess metabolic changes and virulence-specific metabolic responses. These results contribute to our understanding of the pathogenesis of MS and the interactions between the host and mycoplasma.

## 2. Materials and Methods

### 2.1. MS Strain and Culture Conditions

The MS strain was cultured with complete mycoplasma medium, which contained Mycoplasma Broth Base (Frey) medium (BD, Franklin Lakes, NJ, USA), 10% porcine serum, 1% arginine, 1% cysteine, and 1% nicotinamide adenine dinucleotide (NAD), at 37 °C as described previously [5]. The ZX313 and SD2 strains were isolated from swollen leg samples collected in Jiangsu Province and Shandong Province, China, respectively [16]. The titer was measured using a color-changing unit (CCU) assay [17].

### 2.2. Pathogenicity Test

To evaluate the pathogenicity of the ZX313 and SD2 strains, 27 four-week-old SPF (Specific-Pathogen-Free) Leghorn chickens purchased from Xinxing Dahuanong Poultry Eggs Co., Ltd. (Yunfu, China) were randomly divided into three groups of 9 chickens each. Each group of chickens was raised independently in isolation cages. One group was inoculated with 0.5 mL of medium via intramuscular injection in the leg as the negative control, while the other two groups were inoculated with 0.5 mL of the ZX313 or SD2 strain at a concentration of 10^8^ CCU/mL. Two weeks post-infection (wpi), surviving chickens were humanely euthanized, followed by necropsy to observe the lesion severity of their tarsal joints, foot pads, and breastbone. Concurrently, the disease severity of each chicken in each group was scored in accordance with the scoring criteria outlined in Table 1. All chickens that died during the experiment were assigned the highest score. Subsequently, samples of the trachea, tarsal joints, and foot pads were collected for detecting mycoplasma load, while samples of the trachea, foot pads, and breastbone were collected for histopathological analysis. All animal experiments were supervised by the Animal Care Committee of South China Agricultural University (SYXK-2019-0136, 31 March 2019) and carried out in accordance with experimental animal care guidelines.

### 2.3. qPCR Assay

The genomic DNA of tissue samples was extracted using a MolPure^®^ Blood/Cell/Tissue/Bacteria DNA fast Kit (Yeasen, Shanghai, China) following the kit’s instructions and subjected to MS-specific qPCR. Briefly, 10 μL of THUNDERBIRD probe qPCR mix (TOYOBO, Shanghai, China), 0.3 μL of forward primer, 0.3 μL of reverse primer, 0.15 μL of probe, 0.4 μL of 50× ROX reference, 2 μL of template DNA, and ddH_2_O were added to a final volume of 20 μL. The qPCR amplification procedure was as follows: 95 °C for 5 min, followed by 40 cycles of 95 °C for 15 s and 60 °C for 30 s [16,18]. The forward primer sequence was 5′-CTAAATACAATAGCCCAAGGCAA-3′, the reverse primer sequence was 5′-CCTCCTTTCTTACGGAGTACA-3′, and the probe sequence was 5′-FAM-AGCGATACACAACCGCTTTTAGAAT-BHQ1-3′ [19].

### 2.4. Untargeted Metabolomics

Plasma samples were collected and separated one week after the chickens were infected or not infected with the ZX313 and SD2 strains. Given that 3 chickens died in the ZX313-infected group, 6 surviving SPF chickens were randomly selected from each group for metabolomic analysis. A total of 18 samples were stored at −80 °C for subsequent untargeted metabolomics analysis. The collected plasma samples were sent to BGI Co., Ltd. (Shenzhen, China) for processing and identification. All experimental methods and preliminary analysis processes were carried out in accordance with the working standard procedures of BGI Co., Ltd.

### 2.5. Metabolite Extraction

Samples were thawed at 4 °C, of which 100 μL was then pipetted into a 96-well plate. Subsequently, 300 μL of extraction reagent (methanol–acetonitrile–water = 2:2:1) and 10 μL of internal standard were added to the 96-well plate. Then the mixture was vortexed for 1 min and left to stand at −20 °C for 2 h. After centrifugation at 4000 rpm at 4 °C for 20 min, 300 μL supernatant of each mixture was transferred to a freeze-drying concentrator and dried. The residues were re-dissolved in 150 μL of reconstitution reagent (methanol–H_2_O = 1:1), thoroughly mixed for 1 min, and centrifuged at 4000 rpm for 30 min at 4 °C. Then, the supernatant of each reconstitution mixture was transferred to a sample vial. Additionally, 10 μL of supernatant from each sample was combined to prepare a pooled quality control (QC) sample to evaluate the reproducibility and stability of the LC-MS analysis.

### 2.6. UPLC-MS/MS Analysis

A Waters 2D UPLC (Waters, Milford, MA, USA) coupled with a Q Exactive high-resolution mass spectrometer (Thermo Fisher Scientific, Waltham, MA, USA) was used for metabolite separation and detection.

The Waters ACQUITY UPLC BEH C18 column (1.7 μm, 2.1 mm × 100 mm; Waters, USA) was used as the chromatographic column. The mobile phase in the positive mode was composed of 0.1% formic acid (solution A) and acetonitrile (solution B), while the mobile phase in the negative mode was composed of 10 mM ammonium formate (solution A) and acetonitrile (solution B). The gradient elution conditions were as follows: within 0–1 min, the proportion of solution B was 2%; from 1 to 9 min, solution B gradually increased from 2% to 98%; within 9–12 min, solution B remained at 98%; from 12 to 12.1 min, solution B changed from 98% to 2%; from 12.1 to 15 min, solution B remained at 2%. The flow rate was set at 0.35 mL/min and the injection volume was 5 μL.

The Q Exactive mass spectrometer was employed to obtain primary and secondary mass spectrometry data. The full scan range was set from 70 to 1050 *m*/*z* with a resolution of 70,000. The automatic gain control (AGC) target was 3 × 10^6^, and the maximum injection time was 100 ms. The top three precursors were selected for subsequent fragmentation. During fragmentation, the resolution was 17,500, the AGC target was 1 × 10^5^, the maximum injection time was 50 ms, and stepped NCEs of 20 eV, 40 eV, and 60 eV were applied. In the positive and negative ionization modes, the mass spectrometric settings were as follows: the spray voltage was 3.8 kV (positive ionization mode) and −3.2 kV (negative ionization mode); the sheath gas flow rate was 40 arb (arbitrary units); the auxiliary gas flow rate was 10 arb; the auxiliary gas heater temperature was 350 °C; and the capillary temperature was 320 °C.

### 2.7. Data Quality Control and Preprocessing

The data quality after LC-MS/MS analysis was assessed through the reproducibility of QC samples, the evaluation of chromatographic overlap, principal component analysis (PCA), and peak number and peak response intensity differences [20,21].

The raw mass spectrometry data were collected and imported into Compound Discoverer 3.1 (Thermo Fisher Scientific, USA) to export information on the molecular weight (MW), peak area, retention time, and identification results of the compounds. In order to identify metabolites, the main parameters were set as follows: the precursor mass tolerance was less than 5 ppm, the fragment mass tolerance was less than 10 ppm, and the RT tolerance was less than 0.2 min. Subsequently, the results exported from Compound Discoverer 3.1 were imported into metaX for data normalization, batch effect correction, and coefficient of variation (CV) calculation. The metabolites were identified as the combined result of HMDB and KEGG databases. Multivariate statistical analysis (PCA and PLS-DA) and univariate analysis (fold change, FC, and Student’s *t*-test) were combined to screen for differential metabolites between groups. PCA and PLS-DA were used to establish a relationship model between metabolite expression and sample groups, so as to predict the sample group. They were then combined with fold change and Student’s *t*-test to determine the differential metabolites. The pathways were enriched using MetaboAnalyst 6.0 (https://www.metaboanalyst.ca/home.xhtml, accessed on 6 July 2025) with the Gallus gallus (chicken) KEGG pathway database. Random forest analysis was employed to identify differentially expressed metabolites among different groups. Receiver operating characteristic (ROC) curves were utilized to assess the diagnostic accuracy of these metabolites in the validation sets. Diagnostic parameters, including sensitivity and specificity, were determined based on the minimum distance to the top-left corner of the ROC plot.

### 2.8. Image and Statistical Analyses

All statistical graphs were created with GraphPad Prism 6 software. All data are presented as the mean with a 95% confidence interval (CI) from all samples in each group. Statistical significance was tested with the nonparametric *t*-test, and *p* values less than 0.05 were defined as the threshold for statistical significance. *P* values between 0.05 and 0.01 were marked with one asterisk, and those less than 0.01 with two asterisks. Graphs of metabolomics analysis were drawn using the R software package metaX (version 1.4.0) and the CNSknowall website (https://www.cnsknowall.com, accessed on 10 July 2025.).

## 3. Results

### 3.1. Pathogenicity of MS Strains

**Figure 1:**
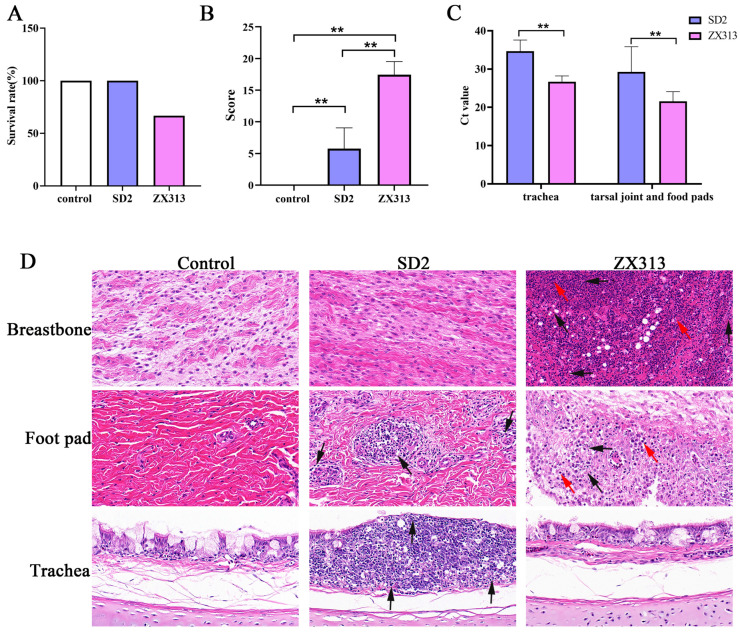
Comparison of pathogenicity between the ZX313 and SD2 strains after 2 weeks of infection. (**A**) Survival rate of chickens. (**B**) Tissue lesion score of chickens. (**C**) The bacterial load results of the trachea and tarsal joint and foot pad detected via qPCR. (**D**) Histopathological analysis of tissues, including breastbone, foot pad, and trachea. Magnification: 200×; *n* = 9. ** indicates *p* < 0.05.

At 7 days post-infection (dpi), chickens infected with the ZX313 strain presented lethargy, disheveled feathers, decreased feed intake, and green fecal excretion. In contrast, chickens in the SD2 strain infection group appeared normal, similarly to the negative control group. After 2 wpi, the mortality of chickens in each group was calculated, and surviving chickens were euthanized and autopsied. Then, diseased tissues were scored following the scoring criteria listed in Table 1. As shown in Figure 1A, all chickens (9/9) in the negative group and SD2-infected group survived, whereas only 66.67% (6/9) of the chickens survived in the ZX313-infected group. The average lesion score was 5.78 and 17.44 for the SD2- and ZX313-infected groups, respectively (Figure 1B). Additionally, the mycoplasma load in the tissues was detected via qPCR. The results reveal that the ZX313 load in the trachea, tarsal joints, and foot pads was significantly greater than that in the SD2 strain (Figure 1C).

The histopathological analysis results are presented in Figure 1D. Compared with the negative control group, the SD2-infected group displayed focal infiltration of inflammatory cells in the mucosal layer of the tracheal tissue, with thinning of the mucosal epithelium (black arrow). There was no significant abnormality in the ZX313-infected group. Pathology of footpad sections revealed that inflammatory cells infiltrated around blood vessels in the SD2-infected group (black arrow). In the ZX313-infected group, the fat layer was necrotic and exfoliated, with a small amount of necrotic cell debris (black arrow) and increased inflammatory cell infiltration (red arrow). Examination of breastbone cyst tissue sections revealed that the ZX313-infected group had extensive necrotic tissues with increased eosinophilic (black arrow) and significant inflammatory cell infiltration, mainly granulocytes and suppurative cells (red arrow). There was no significant difference between the SD2-infected group and the negative control group. Overall, the ZX313 strain exhibited strong pathogenicity to joint tissues but had minimal impact on the respiratory system, whereas the SD2 strain tended to affect the respiratory system, although its pathogenicity was weak. Thus, we conclude that MS infection mainly causes inflammatory responses in joint tissues, and the pathogenicity of strains is positively correlated with the degree of induced inflammation.

### 3.2. Overview of Metabolic Changes After MS Infection

**Figure 2:**
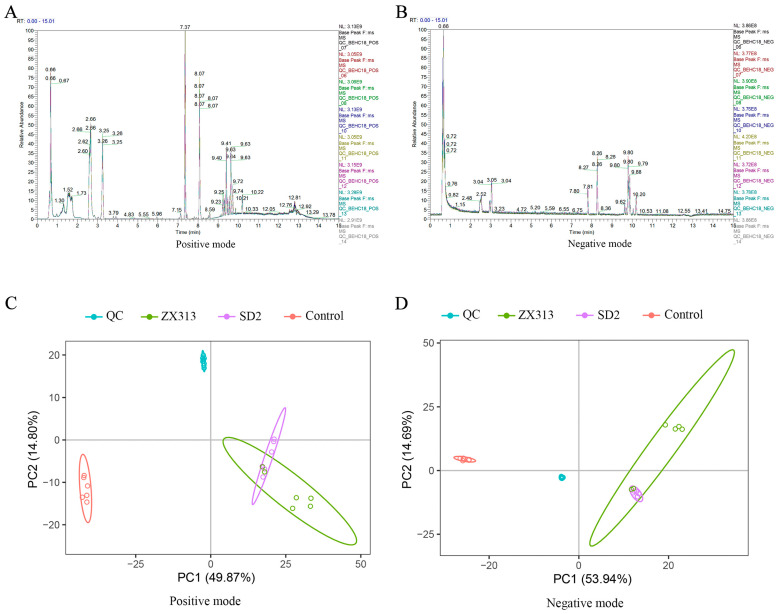
BPCs of all QC samples and PCA plot of untargeted metabolomics in MS-infected and control SPF chickens. BPCs of QC samples in positive (**A**) and negative mode (**B**). PCA plot of all samples in positive (**C**) and negative (**D**). Each dot represents an individual sample. *n* = 6.

To characterize the metabolic responses to MS infection, a non-target LC-MS-based metabolomics analysis was conducted on plasma samples collected from the following groups of SPF chickens: the control, those infected with highly pathogenic strain ZX313, and those infected with low-pathogenic strain SD2. The base peak chromatograms (BPCs) of all QC samples indicate that the instrument was in good condition and that the signal was stable throughout the entire sample detection and analysis process (Figure 2A,B). PCA was used to investigate the overall distribution of the samples in each group and the stability of the entire analysis process. Better aggregation of QC samples indicates greater instrument stability and better repeatability of the acquired data. Overall, there was a noticeable distinction between the groups and the QC samples (Figure 2C,D), and the ZX313- and SD2-infected groups partially overlapped. This suggests that the host metabolic disorders induced by the two MS strains are both consistent and specific.

**Figure 3:**
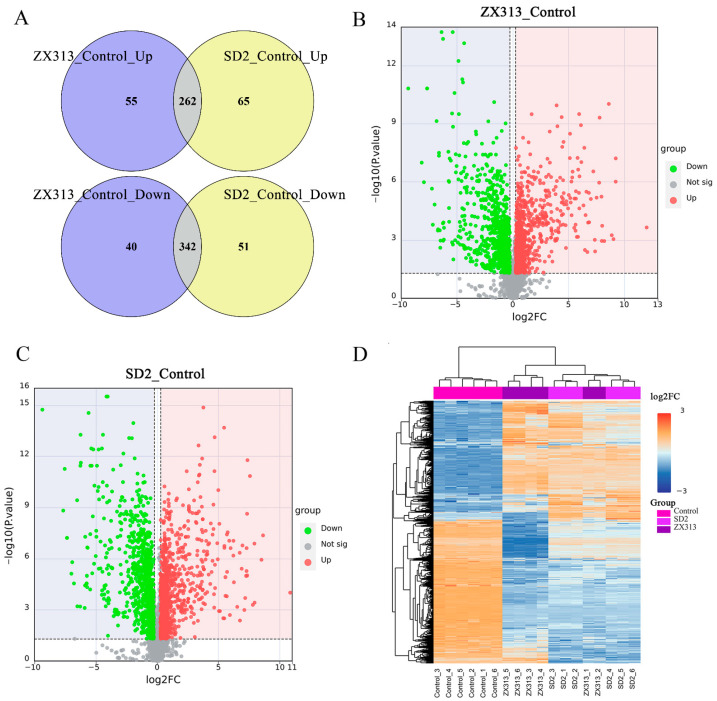
Multivariate analysis of metabolic profiles induced by MS infection. (**A**) Venn diagrams show the number of upregulated and downregulated SDMs in the ZX313- and SD2-infected groups. Volcano plots of SDMs in the ZX313-infected (**B**) and SD2-infected (**C**) groups. Each point represents a metabolite. Red, upregulated; green, downregulated; gray, nonsignificant. (**D**) Heatmap of hierarchical clustering analysis of SDMs in different groups. Log2FC, log2(fold change).

In total, we obtained 3129 metabolites (RSD ≤ 30%) from the untargeted metabolomics data, but only 1061 were identified. In this study, significantly differentially abundant metabolites (SDMs) were identified using VIP ≥ 1, fold change ≥ 1.2 or ≤0.83, and *p* value < 0.05. Significant differences were observed in the types and abundances of metabolites induced with different virulent MS strains in SPF chickens (Figure 3). Compared with the control group, 699 (317 upregulated and 382 downregulated) and 720 (327 upregulated and 393 downregulated) SDMs were identified in the ZX313- and SD2-infected groups, respectively (Figure 3A). A volcano plot visually revealed that the abundances of host metabolites were variably upregulated and downregulated following MS strain infection in SPF chickens (Figure 3B,C).

To elucidate the host metabolic characteristics of SPF chickens infected with MS strains, the untargeted metabolomics data underwent hierarchical clustering. As shown in Figure 3D, after infection with the MS strains, the metabolic profiles of SPF chickens exhibited varying degrees of regulation. Although there were some differences in metabolite expression profiles among different chickens within the infection groups, the trend of the differential expression of metabolites in the host induced by virulent and attenuated MS strains was nearly consistent compared with that in the control group.

### 3.3. MS Infection Disrupts Host Metabolism

**Figure 4:**
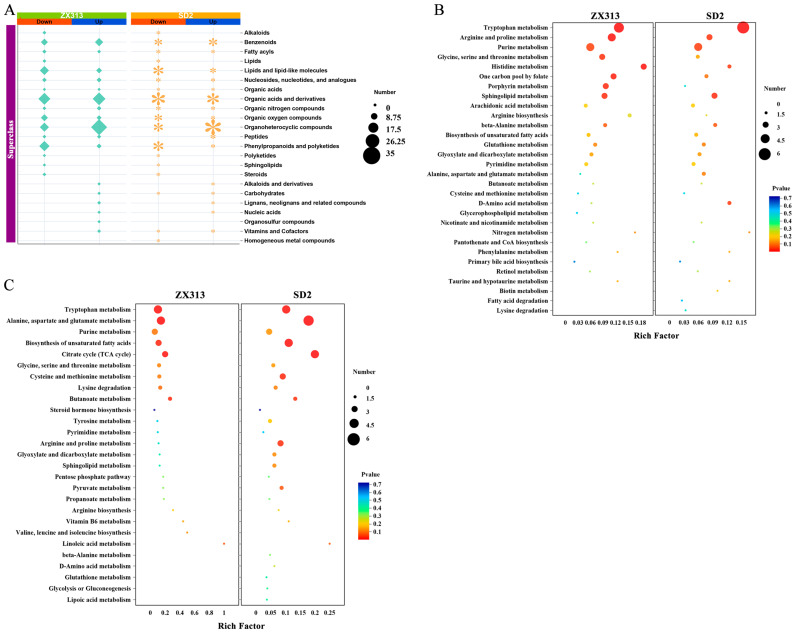
Enrichment analysis of SDMs after MS infection. (**A**) Superclass of SDMs in ZX313- and SD2-infected groups. KEGG pathway analysis of upregulated SDMs (**B**) and downregulated SDMs (**C**) in ZX313- and SD2-infected groups.

To gain a clearer understanding of the patterns of metabolite changes in SPF chickens following MS infection, upregulated and downregulated SDMs were analyzed, respectively. As shown in Figure 3A, there were 317 upregulated SDMs and 382 downregulated SDMs in the ZX313 infected group, and 327 upregulated SDMs and 393 downregulated SDMs in the SD2 infected group. Overall, 262 upregulated and 342 downregulated SDMs were identified in both infection groups (Figure 3A). The metabolite classification results show that the SDMs in the ZX313- and SD2-infected groups were each categorized into 22 super-classes. The five most abundant superclasses were organic acids and derivatives, organoheterocyclic compounds, benzenoids, lipids and lipid-like molecules, and phenylpropanoids and polyketides (Figure 4A). Subsequently, these SDMs were annotated using the KEGG and HMDB databases. KEGG pathway analysis revealed that, among the upregulated metabolites, tryptophan metabolism, histidine metabolism, arginine and proline metabolism, sphingolipid metabolism, and purine metabolism pathways were significantly enriched (*p* < 0.05) in both infected groups. In contrast, the one-carbon pool by folate, porphyrin metabolism, and glycine, serine, and threonine metabolism pathways were specifically enriched (*p* < 0.05) in the ZX313-infected group, whereas the D-amino acid metabolism pathway was specifically enriched (*p* < 0.05) in the SD2-infected group (Figure 4B). Among the downregulated metabolites, alanine, aspartate and glutamate metabolism, tryptophan metabolism, citrate cycle (TCA cycle), butanoate metabolism, and biosynthesis of unsaturated fatty acids pathways were significantly enriched (*p* < 0.05) in both infected groups, while cysteine and methionine metabolism and arginine and proline metabolism pathways were specifically enriched (*p* < 0.05) in the SD2-infected group (Figure 4C). These results demonstrate that MS infection primarily disrupts the host’s amino acid metabolism, lipid metabolism, carbohydrate metabolism, and metabolism of cofactor and vitamin pathways.

The information on the top 20 annotated common SDMs between the ZX313- and SD2-infected groups is shown in Table 2. Notably, among these top 20 SDMs, nearly half are associated with intestinal microbiota and their metabolic processes. Specifically, deoxycholate, glycoursodeoxycholic acid, and beta-muricholate are distinct bile acid derivatives. As amphipathic molecules are synthesized in the liver, they exert biological functions in the intestine and participate in enterohepatic circulation, with core roles in regulating lipid digestion and absorption, intestinal microecological homeostasis, and hepatic metabolism [22]. The downregulated abundances of these bile acids suggest that MS infection may induce hepatic dysfunction, impaired cholesterol synthesis in chickens, and bile acid metabolism disorders, further caused by intestinal microbiota dysbiosis. Additionally, hippurate, 3-(2-hydroxyphenyl)propanoate, and DL-4-hydroxyphenyllactic acid are all metabolites of aromatic amino acids produced by intestinal microbiota [23]. The increased abundance of hippurate and decreased abundance of 2-HPPA and DL-HPLA indicate elevated abundance of E. coli in the intestinal microbiota and exacerbated intestinal inflammation [24] following MS infection.

### 3.4. Metabolites and Pathway Analysis with Different Virulent MS Infections

**Figure 5:**
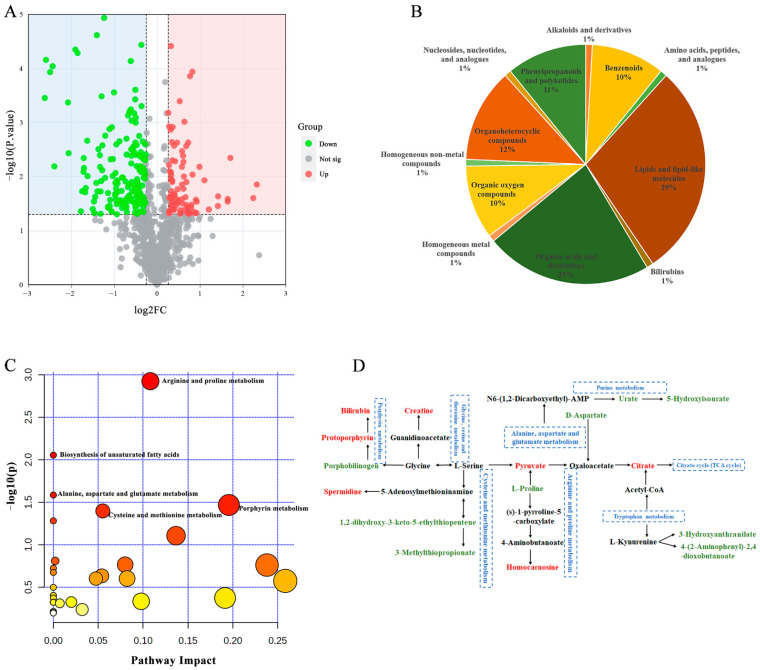
Metabolite and pathway analysis between ZX313- and SD2-infected groups. (**A**) Volcano plots, (**B**) classification, (**C**) pathway enrichment analysis and (**D**) metabolic pathways of SDMs between the two groups. The significantly different metabolic pathways are marked; metabolites colored in red or green represent significantly higher or lower concentrations in the ZX313-infected group compared to the SD2-infected group, respectively (*p* < 0.05).

According to the differential metabolites, we analyzed the regulatory changes and levels of detected metabolites in the plasma of SPF chickens following infection with MS strains of different virulence. Compared with the SD2-infected group, the ZX313-infected group exhibited a significantly altered abundance of 301 metabolites (fold change ≥ 1.2 or ≤0.83, *p* value < 0.05), of which 187 were downregulated and 114 were upregulated (Figure 5A). These SDMs were mainly classed as lipids and lipid-like molecules, and organic acids and derivatives (Figure 5B). KEGG pathway enrichment revealed that these SDMs were involved in 13 predicted pathways with hit values ≥ 2, and 5 of these pathways were recognized as main metabolic pathways with *p* value < 0.05, including arginine and proline metabolism, biosynthesis of unsaturated fatty acids, alanine, aspartate and glutamate metabolism, porphyrin metabolism, and cysteine and methionine metabolism (Figure 5C). The possible metabolic pathways altered after ZX313 and SD2 infection are shown in Figure 5D. Compared with the SD2-infected group, the ZX313-infected group exhibits a greater tendency to enhance the metabolism of amino acids (excluding tryptophan, methionine, and aspartic acid), which is mainly reflected in the increased abundances of terminal compounds in multiple amino acid metabolic pathways. Additionally, the purine metabolic pathway is downregulated, which ultimately results in a significant decrease in the contents of its end products, urate and 5-hydroxyisourate (Figure 5D). Table 3 shows information on the top 20 annotated SDMs in the ZX313-infected group compared to the SD2-infected group. Metabolomics studies have demonstrated that *Mycoplasma pneumoniae* (MP) infection can increase the levels of triglycerides and cholesterol in the host, while activating the fatty acid β-oxidation pathway to meet its own energy requirements [25]. In the present study, MS infection significantly altered the host’s lipid metabolic pathways (Figure 4). Furthermore, compared with the SD2-infected group, the abundances of hexanoylcarnitine and 9-oxo-10(e),12(e)-octadecadienoic acid were significantly upregulated in the ZX313-infected group. Specifically, hexanoylcarnitine, a key carrier molecule for fatty acid β-oxidation, transports medium-chain fatty acids from the cytoplasm to the mitochondrial matrix, where these fatty acids are converted into acetyl-CoA through the β-oxidation pathway. In contrast, 9-oxo-10(e),12(e)-octadecadienoic acid activates peroxisome proliferator-activated receptor α (PPARα), which upregulates the expression of carnitine palmitoyltransferase 1 (CPT1)—the rate-limiting enzyme of fatty acid β-oxidation. This activation indirectly promotes the transport and oxidation of medium-chain fatty acids mediated by hexanoylcarnitine [26]. Together, these two metabolites synergistically enhance the efficiency of fatty acid catabolism in the host. Thus, the activation level of the host fatty acid β-oxidation pathway may also be associated with the virulence of MS strains.

### 3.5. Metabolites in Relation to the Severity of MS Infections

**Figure 6:**
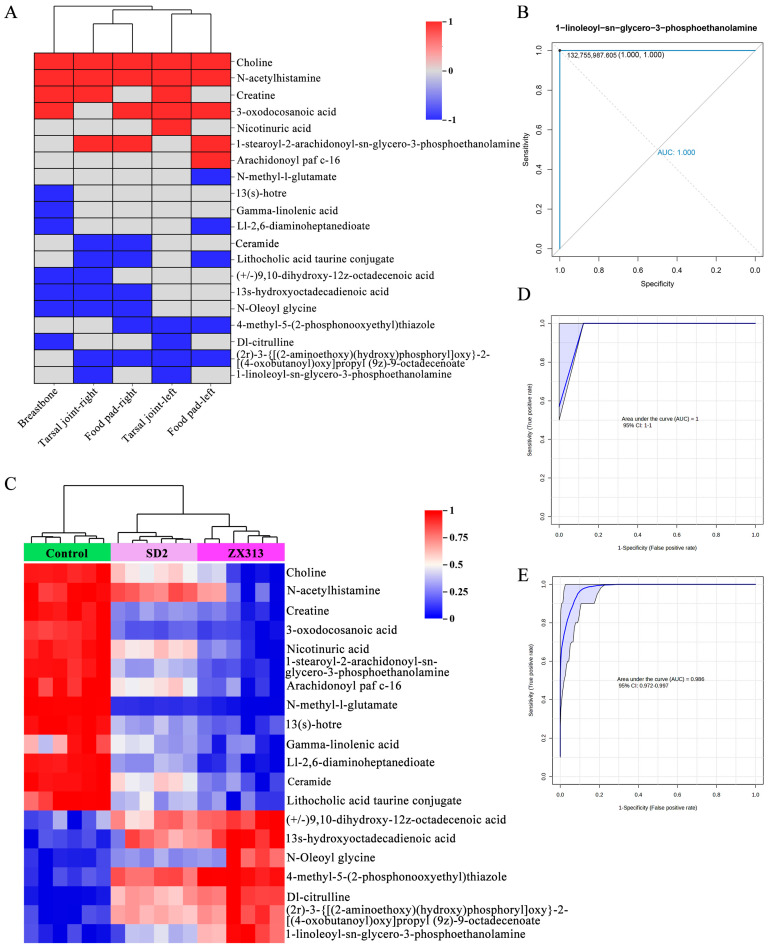
Metabolites in relation to the severity of MS infections. (**A**) Correlation analysis between plasma SDMs in the ZX313- and SD2-infected groups and various joint lesion indices of SPF chickens. (**B**) The 1-linoleoyl-sn-glycero-3-phosphoethanolamine in plasma achieved an AUC of 1 for distinguishing MS infections from controls. (**C**) Heatmap of the expression of SDMs associated with clinical symptom severity. (**D**) The combination of the 20-metabolite panel in plasma achieved an AUC of 1 for distinguishing MS infections and controls and (**E**) an AUC of 0.986 for distinguishing ZX313- and SD2-infected chickens.

After being infected with the MS strain isolated in this study, ZX313, SPF chickens developed significant swelling in the tarsal joints, foot pads, and breastbones, accompanied by a large amount of inflammatory mucus and cheesy material, and even death. In contrast, after infection with the SD2 strain, the SPF chickens exhibited milder clinical symptoms, with only a small amount of inflammatory mucus present in the joints. To identify metabolites linked to the severity of MS infection, we further analyzed pathologically relevant metabolites in the ZX313-infected group compared to the SD2-infected group. First, we evaluated the correlations between plasma SDMs in the two groups and various joint lesion indices of SPF chickens. Among these SDMs, the top 20 that exhibited the highest significant correlations with different joint lesions are presented in Figure 6A. Receiver operating characteristic (ROC) curves (with corresponding area under the curve, AUC, values calculated) were generated for each SDM to assess their response to MS infection. The AUC values for all individual metabolites are presented in Figure S1, with Figure 6B showing a representative example. All identified metabolites demonstrate potential as biomarkers for distinguishing MS infections with an AUC ≥ 0.950. The expression levels of these 20 SDMs across different samples are illustrated in Figure 6C. It can be observed that as the pathogenicity of MS strains increased, the abundances of these metabolites in different groups exhibited corresponding directional upward or downward trends. In this study, these 20 SDMs were considered an integrated panel, and the response of this panel to MS infection, as well as to infections by MS strains with varying pathogenicity, was further evaluated. As shown in Figure 6D,E, the AUC values of this metabolite panel were 1 and 0.986, respectively, indicating that the 20-metabolite panel exhibits high accuracy in distinguishing between the MS-infected group and the control group, as well as between MS strains with varying virulence. These results highlight the potential of the aforementioned 20-metabolite panel as a reliable tool for evaluating the severity and presence of MS infection.

## 4. Discussion

MS is mostly a recessive infection and difficult to control, which makes MS one of the pathogens of primary concern for the global poultry industry. In the absence of coinfection with other pathogens, there are few reports on severe pathogenesis caused by MS infection alone. In this study, a highly virulent strain of MS was identified, which can even cause the death of chickens. The minimal lethal dose of the ZX313 strain is lower than 10^6^ CCU/mL. Compared with the less virulent SD2 strain, ZX313 infection causes a more severe inflammatory response in joint tissues, as shown in histopathological analysis results. In order to further explore the pathogenic mechanisms of different virulent MS strains and the metabolites related to the severity of post-infection morbidity, we performed untargeted metabolomics analysis on plasma samples of SFP chickens infected with different virulent MS strains.

Mycoplasmas apparently lost almost all genes involved in the biosynthesis of amino acids, fatty acids, cofactors, and vitamins, and therefore, they depend on the host microenvironment to supply the full spectrum of biochemical precursors required for the biosynthesis of macromolecules [27]. In our study, MS infection leads to metabolic disorders in the host, mainly manifested as amino acid metabolism disorders, nucleotide metabolism disorders, lipid metabolism disorders, and cofactors and vitamins metabolism disorders. According to the reports, MP infection alters metabolites in several pathways related to amino acid, nucleotide, energy, and lipid metabolism [25,28]. *Mycoplasma hyopneumoniae* (*M. hyopneumoniae*) infection altered the serum levels of select amino acids and fatty acids in a time-dependent manner [29]. Based on the above research and our findings, we can conclude that mycoplasma infection leads to systemic metabolic disorders in the host, with amino acid and lipid metabolism playing a crucial role in mycoplasma infection.

Amino acids are essential nutrients for life. SDMs related to amino acid metabolism were detected in both ZX313- and SD2-infected plasma samples, suggesting that amino acid metabolism plays a crucial role in MS infection. Amino acids are vital for protein transport. During MS infection, proteins are continuously decomposed into amino acids, which not only provide the amino acids necessary for the proliferation of MS in the host but also supply energy for the host body through oxidative metabolism and carbohydrate consumption. This process may lead to constant depletion of the host body, which eventually manifests as poor mental performance and body wasting. Furthermore, enhanced amino acid metabolism generates key molecules with pro-inflammatory or anti-inflammatory activity, which directly regulate inflammatory pathways. In this study, following MS infection, the abundance of kynurenic acid was significantly increased among the top 20 SDMs. As a key intermediate in the tryptophan metabolic pathway, the increased abundance of kynurenic acid indicates the presence of an inflammatory response in the organism [30]. This response promotes the preferential shunting of tryptophan to kynurenine pathway metabolism, ultimately leading to increased kynurenic acid production. Studies have shown that the OppF protein is responsible for the uptake of peptides and sugars, and plays a role in the pathogenicity and immunogenicity of MS [14]. However, this study did not involve expression analysis at the genomic level of MS strains; for the two MS strains with different pathogenicity, the expression levels of the OppF protein and its role in the pathogenic process remain unclear. Therefore, integrated analysis of genomics, proteomics, and metabolomics may be more conducive to elucidating the regulatory mechanisms underlying differences in MS pathogenicity.

Mycoplasmas lack the de novo synthesis pathway for pyrimidine and purine synthesis [31]. Consequently, they can only synthesize the nucleotides essential for their growth through direct uptake of exogenous nucleotides or via salvage pathways. In the SDMs post-infection with ZX313 and SD2, nucleotide metabolism-related metabolites were enriched. Adenosine is the direct precursor of adenine nucleotides, and guanine is the base precursor of guanine nucleotides; 2′-deoxyguanosine and 2′-deoxyinosine are deoxy purine nucleosides required for DNA synthesis. Thymine is a pyrimidine base, and thymidine is a thymidine nucleoside—both serve as precursors of deoxythymidine triphosphate (dTTP), a pyrimidine nucleotide specific to DNA. The upregulation of these metabolites suggests that the nucleotide salvage synthesis pathway is activated, directly indicating an increased demand for DNA synthesis [32], while nucleotide catabolic products inosine and hypoxanthine and their end product uric acid all exhibited decreased abundances in the MS-infected groups [33]. This collectively suggests that the purine catabolic pathway is inhibited. These results indicate that following MS infection, the host’s nucleotide catabolic pathway is inhibited, while the nucleotide salvage synthesis pathway is commonly activated. In a study on MP infection, inosine was significantly associated with disease severity [25]. However, in this study, there was no significant difference in the inosine content between the two infected groups.

Mycoplasmas acquire specific lipids from the environment and thereby influence lipid metabolism [34]. Nair et al. detected porcine plasma metabolites and reported significantly elevated concentrations of total free fatty acids (FFAs) and individual FFAs (myristic, palmitoleic, oleic, linoleic acids) during early *M. hyopneumoniae* infection, with the infection altering plasma levels of selected amino acids and fatty acids in a time-dependent manner [29]. Similarly, metabolomics analysis of MP infection showed altered host glycerophospholipid and sphingolipid metabolism, with differentially expressed lipid metabolites potentially linked to disease severity [28]. However, in our study, in the KEGG pathway enrichment analysis, only a small number of SDMs were involved in lipid metabolism-related pathways after MS infection. Nevertheless, consistent with the metabolomic studies on SPF laying hens infected with reproductive system-tropic MS strains [15], MS infection in chickens significantly affects the host’s Sphingolipid metabolism pathway and steroid hormone biosynthesis pathway. Lipids play a critical role in the formation and structural integrity of mycoplasma membranes. In studies on metabolic reversal caused by mutations in live MS vaccine strains and the mechanism of magnolol against MS, both highlight the importance of lipids in the complete life activities of MS [14,35]. However, the metabolites of mycoplasma itself may be largely masked by host metabolites, which results in the inevitable loss of some metabolic data closely associated with mycoplasma metabolism during the analysis of host metabolites.

The correlation analysis between metabolites and clinical symptoms found that lipids and lipid-related substances, choline, ceramide,1-stearoyl-2-arachidonoyl-sn-glycero-3-phosphoethanolamine, (2r)-3-{[(2-aminoethoxy)(hydroxy)phosphoryl]oxy}-2-[(4-oxobutanoyl)oxy]propyl (9z)-9-octadecenoate, and 1-linoleoyl-sn-glycero-3-phosphoethanolamine distinguish MS infection status and strain virulence differences with high accuracy. 1-stearoyl-2-arachidonoyl-sn-glycero-3-phosphoethanolamine, (2r)-3-{[(2-aminoethoxy)(hydroxy)phosphoryl]oxy}-2-[(4-oxobutanoyl)oxy]propyl, (9z)-9-octadecenoate, and 1-linoleoyl-sn-glycero-3-phosphoethanolamine are phosphatidylethanolamines. They participate in membrane phospholipid remodeling and play critical roles in mycoplasma proliferation and cellular signal regulation post-infection. Ceramides are a major structural component of sphingolipids. Various sphingolipids and their components are critical for regulating cellular signals (e.g., cell growth, inflammation, and cell death) [36]. Choline, a nitrogen-containing water-soluble vitamin-like substance, is involved in the key synthesis of phosphatidylcholine, sphingomyelin, and the neurotransmitter acetylcholine. It also promotes the maintenance of cell membrane structural integrity and cellular signal transduction [37]. One limitation of this study is that during the untargeted metabolomics analysis, no dedicated omics analysis was conducted for lipid components, which results in a lack of comprehensive understanding of the role of lipids in MS infection and post-infection disease severity.

## 5. Conclusions

In this study, we investigated changes in the plasma metabolome of SPF chickens infected with two MS strains of differing virulence. MS infection induces abnormal expression of numerous host metabolites, particularly those involved in amino acid metabolism, nucleotide metabolism, and lipid metabolism. Additionally, we analyzed the metabolic differences caused by infection with MS strains of varying virulence, as well as biomarker panels for distinguishing MS infection and the virulence of infected strains. Thus, this study provides valuable insights for further investigating the mechanisms of MS infection and the underlying causes of differences in MS pathogenicity.

## Figures and Tables

**Table 1:** Tissue lesion scoring criteria.

Tissue	Lesion Degree	**Score**
tarsal joint, foot pad, and breastbone	no obvious swelling, no inflammatory mucus, no cheesy material	0
	no obvious swelling, a small amount of inflammatory mucus, no cheesy material	1
	slight swelling, large amount of inflammatory mucus, no cheesy material	2
	obviously swollen, a large amount of inflammatory mucus, and a small amount of cheesy material	3
	obviously swollen, a large amount of inflammatory mucus and cheesy material	4

**Table 2:** Common SDMs in ZX313- and SD2-infected groups annotated using the KEGG and HMDB databases.

Number	Metabolite_Name	Metabolite Formula	Actual.RT	Database Annotation		Super Class	ZX313 Infection		SD2 Infection	
				KEGG	HMDB		Fold Change	*p* Value	Fold Change	*p* Value
1	Deoxycholate	C_24_H_40_O_4_	8.783	C04483	HMDB0000626	Steroids	0.0120	6.95 × 10^−13^	0.0135	7.06 × 10^−13^
2	8-oxoguanine	C_5_H_3_N_5_O_2_	4.329	/	HMDB0041820	Organoheterocyclic compounds	28.2861	4.14 × 10^−6^	51.9779	9.03 × 10^−7^
3	Glycoursodeoxycholic acid	C_26_H_43_NO_5_	8.326	/	HMDB0000708	Lipids and lipid-like molecules	0.0231	1.72 × 10^−12^	0.0641	8.30 × 10^−12^
4	Hippurate	C_9_H_9_NO_3_	3.821	C01586	HMDB0000714	Benzenoids	24.1298	2.14 × 10^−3^	29.2069	2.02 × 10^−4^
5	Triethanolamine	C_6_H_15_NO_3_	0.697	C06771	HMDB0032538	Organic nitrogen compounds	24.4627	9.16 × 10^−5^	17.8228	2.34 × 10^−6^
6	beta-Muricholate	C_24_H_40_O_5_	8.288	C17726	HMDB0000415	Lipids and lipid-like molecules	0.0380	8.91 × 10^−11^	0.0639	7.24 × 10^−11^
7	Lupulone	C_26_H_38_O_4_	8.907	C10706	HMDB0030041	Organic oxygen compounds	0.0488	5.26 × 10^−15^	0.0469	5.14 × 10^−15^
8	Gluconolactone	C_6_H_10_O_6_	0.624	C00198	HMDB0000150	Organic oxygen compounds	0.0433	4.03 × 10^−11^	0.0769	5.33 × 10^−11^
9	Patuletin	C_16_H_12_O_8_	0.604	C10118	HMDB0030802	Phenylpropanoids and polyketides	0.0584	1.30 × 10^−11^	0.0795	1.93 × 10^−11^
10	Dodecanedioic acid	C_12_H_22_O_4_	5.978	C02678	HMDB0000623	Lipids and lipid-like molecules	16.0433	7.76 × 10^−4^	11.5431	1.27 × 10^−3^
11	Kynurenic acid	C_10_H_7_NO_3_	3.08	C01717	HMDB0000715	Organoheterocyclic compounds	8.0333	1.94 × 10^−3^	13.1842	1.76 × 10^−3^
12	3-(2-hydroxyphenyl)propanoate	C_9_H_10_O_3_	2.857	C01198	HMDB0033752	Phenylpropanoids and polyketides	0.2075	1.74 × 10^−8^	0.0662	4.62 × 10^−16^
13	Hexanoic acid	C_6_H_12_O_2_	3.29	C01585	HMDB0000535	Lipids and lipid-like molecules	0.1500	1.21 × 10^−2^	0.1222	1.01 × 10^−2^
14	Corticosterone	C_21_H_30_O_4_	6.91	C02140	HMDB0001547	Lipids and lipid-like molecules	0.1475	1.14 × 10^−13^	0.1267	2.39 × 10^−13^
15	Daidzein	C_15_H_10_O_4_	5.816	C10208	HMDB0003312	Phenylpropanoids and polyketides	0.1408	7.18 × 10^−14^	0.1640	6.81 × 10^−12^
16	Phenyllactate	C_9_H_10_O_3_	3.029	C05607	HMDB0000748	Phenylpropanoids and polyketides	0.1396	6.07 × 10^−15^	0.1835	1.05 × 10^−16^
17	Acetophenone	C_8_H_8_O	3.031	C07113	HMDB0033910	Organic oxygen compounds	0.1396	6.07 × 10^−15^	0.1835	1.05 × 10^−16^
18	Sinapinic acid	C_11_H_12_O_5_	4.742	C00482	HMDB0032616	Phenylpropanoids and polyketides	0.1066	1.47 × 10^−7^	0.3501	2.98 × 10^−9^
19	Dambonitol	C_8_H_16_O_6_	3.051	/	HMDB0033942	Organic oxygen compounds	6.40533	2.10 × 10^−6^	4.6094	1.98 × 10^−4^
20	Dl-4-hydroxyphenyllactic acid	C_9_H_10_O_4_	1.255	C03672	HMDB0000755	Phenylpropanoids and polyketides	0.1834	4.17 × 10^−8^	0.1844	3.89 × 10^−8^

**Table 3:** The information on the top 20 annotated SDMs in the ZX313-infected group compared to the SD2-infected group.

Number	Metabolite_Name	Metabolite Formula	Actual.RT	Database Annotation		Super Class	Fold Change	*p*-Value
				**KEGG**	**HMDB**			
1	Thromboxane b2	C_20_H_34_O_6_	6.914	C05963	HMDB0003252	Lipids and lipid-like molecules	0.1661	1.97 × 10^−6^
2	Sinapinic acid	C_11_H_12_O_5_	4.742	C00482	HMDB0032616	Phenylpropanoids and polyketides	0.3045	2.60 × 10^−3^
3	3-(2-hydroxyphenyl)propanoate	C_9_H_10_O_3_	2.857	C01198	HMDB0033752	Phenylpropanoids and polyketides	3.1363	1.53 × 10^−2^
4	4-hydroxy-1h-indole-3-acetonitrile	C_10_H_8_N_2_O	3.36	/	HMDB0038462	Organoheterocyclic compounds	0.3254	1.35 × 10^−3^
5	1,2-dihydroxy-3-keto-5-methylthiopentene	C_6_H_10_O_3_S	2.506	C15606	HMDB0012134	Organic oxygen compounds	0.3538	1.04 × 10^−2^
6	Nicotinuric acid	C_8_H_8_N_2_O_3_	3.02	C05380	HMDB0003269	Organic acids and derivatives	2.6766	1.86 × 10^−2^
7	Methyl jasmonate	C_13_H_20_O_3_	8.515	C11512	HMDB0036583	Fatty acyls	0.3779	6.14 × 10^−6^
8	20-hydroxy-(5z,8z,11z,14z)-eicosatetraenoic acid	C_20_H_32_O_3_	8.881	C14748	HMDB0005998	Lipids and lipid-like molecules	0.4255	5.40 × 10^−7^
9	Hexanoylcarnitine	C_13_H_25_NO_4_	4.525	/	HMDB0000705	Fatty Acyls	2.3119	1.29 × 10^−2^
10	4-vinylphenol sulfate	C_8_H_8_O_4_S	4.719	/	HMDB0062775	Organic acids and derivatives	0.4328	4.82 × 10^−5^
11	Androstanolone	C_19_H_30_O_2_	8.814	C03917	HMDB0002961	Lipids and lipid-like molecules	0.4824	8.10 × 10^−5^
12	Pyrogallol-2-o-glucuronide	C_12_H_14_O_9_	2.785	/	HMDB0060017	Organic oxygen compounds	0.4990	8.63 × 10^−4^
13	Isoliquiritigenin	C_15_H_12_O_4_	6.348	C08650	HMDB0037316	Phenylpropanoids and polyketides	1.9874	1.12 × 10^−2^
14	3-indoxyl sulphate	C_8_H_7_NO_4_S	3.067	/	HMDB0000682	Organic acids and derivatives	0.5096	7.50 × 10^−3^
15	8-oxoguanine	C_5_H_3_N_5_O_2_	4.329	/	HMDB0041820	Organoheterocyclic compounds	0.5442	1.88 × 10^−3^
16	Gluconolactone	C_6_H_10_O_6_	0.624	C00198	HMDB0000150	Organic oxygen compounds	0.5630	4.03 × 10^−3^
17	4-(2-aminophenyl)-2,4-dioxobutanoic acid	C_10_H_9_NO_4_	3.813	C01252	HMDB0000978	Organic oxygen compounds	0.5689	2.60 × 10^−3^
18	9-oxo-10(e),12(e)-octadecadienoic acid	C_18_H_30_O_3_	8.721	C14766	HMDB0004669	Lipids and lipid-like molecules	1.6621	4.87 × 10^−2^
19	Glycitein	C_16_H_12_O_5_	5.949	C14536	HMDB0005781	Phenylpropanoids and polyketides	1.6371	9.43 × 10^−3^
20	N-acetylproline	C_7_H_11_NO_3_	3	/	HMDB0094701	Organic acids and derivatives	0.6249	1.40 × 10^−3^

## Data Availability

The metabolomics data of MS strains ZX313 and SD2 are available from MetaboLights with identification number MTBLS11832 (https://www.ebi.ac.uk/metabolights/MTBLS11832) (accessed on 15 December 2024). The raw data of the pathogenicity test have been uploaded to the Figshare database with DOI: 10.6084/m9.figshare.28218617.

## Supplementary Materials

The following supporting information can be downloaded at: https://www.mdpi.com/article/10.3390/microorganisms13112427/s1, Figure S1. The AUC values of each metabolite in Figure 6A for distinguishing MS infections from controls.

## Abbreviations

The following abbreviations are used in this manuscript:

MS	*Mycoplasma synoviae*
SDM	Significantly differentially abundant metabolites
NAD	Nicotinamide adenine dinucleotide
CCU	Color-changing unit
SPF	Specific pathogen-free
WPI	Weeks post-infection
QC	Quality control
AGC	Automatic gain control
PCA	Principal component analysis
ROC	Receiver operating characteristic
BPC	Base peak chromatograms
MP	*Mycoplasma pneumoniae*
PPARα	Peroxisome proliferator-activated receptor α
CPT1	Carnitine palmitoyltransferase 1
