# Supplementary figures and images for: Potential Biomarkers and Underlying Pathogenesis of Mycoplasma synoviae Infection: Insights from Metabolomics Analysis
Source: Microorganisms. 2025 Oct 23;13(11):2427. doi: 10.3390/microorganisms13112427 (PMC12654258; doi:10.3390/microorganisms13112427)

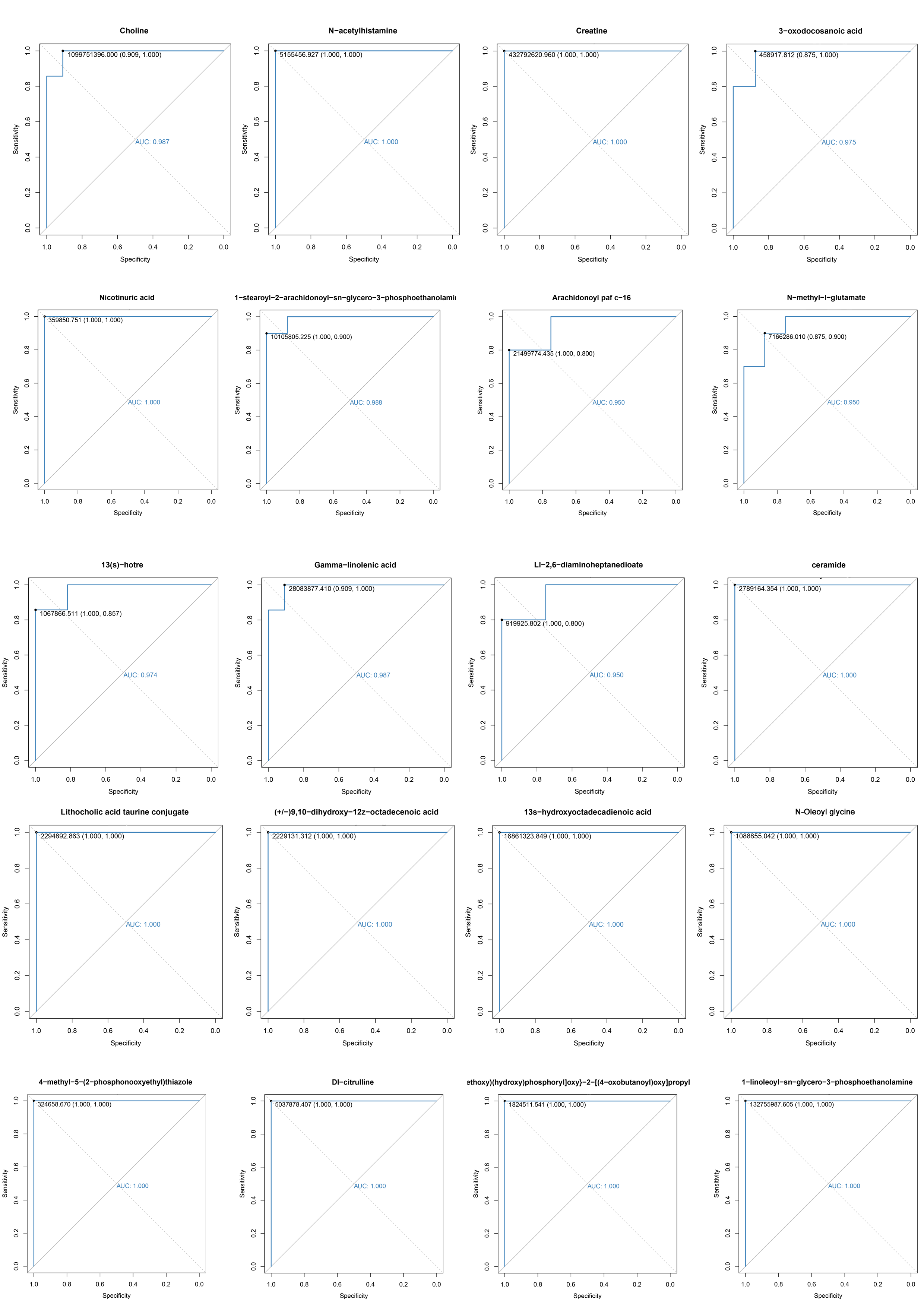

Supplement: Supplementary file 1 [file microorganisms-13-02427-s001.zip › Figure S1.tif]
